# Emergency myelopoiesis contributes to immune cell exhaustion and pulmonary vascular remodelling

**DOI:** 10.1111/bph.14945

**Published:** 2020-02-04

**Authors:** Chunhua Fu, Yuanqing Lu, Mason A. Williams, Mark L. Brantly, Corey E. Ventetuolo, Laurence M. Morel, Borna Mehrad, Edward W. Scott, Andrew J. Bryant

**Affiliations:** ^1^ Division of Pulmonary, Critical Care, and Sleep Medicine, Department of Medicine, College of Medicine University of Florida Gainesville Florida; ^2^ Division of Pulmonary, Critical Care and Sleep Medicine Alpert Medical School of Brown University Providence Rhode Island; ^3^ Department of Pathology, Immunology and Laboratory Medicine University of Florida College of Medicine, University of Florida Gainesville Florida; ^4^ Department of Molecular Genetics and Microbiology University of Florida College of Medicine, University of Florida Gainesville Florida

## Abstract

**Background and Purpose:**

Pulmonary hypertension (PH) secondary to chronic lung disease (World Health Organization Group 3 PH) is deadly, with lung transplant being the only available long‐term treatment option. Myeloid‐derived cells are known to affect progression of both pulmonary fibrosis and PH, although the mechanism of action is unknown. Therefore, we investigated the effect of myeloid cell proliferation induced by emergency myelopoiesis on development of PH and therapy directed against programmed death‐ligand 1 (PD‐L1), expressed by myeloid cells in prevention of pulmonary vascular remodelling.

**Experimental Approach:**

LysM.Cre‐DTR (“mDTR”) mice were injected with bleomycin (0.018 U·g^−1^, i.p.) while receiving either vehicle or diphtheria toxin (DT; 100 ng, i.p.) to induce severe PH. Approximately 4 weeks after initiation of bleomycin protocol, right ventricular pressure measurements were performed and tissue samples collected for histologic assessment. In a separate experiment, DT‐treated mice were given anti‐PD‐L1 antibody (αPD‐L1; 500 μg, i.p.) preventive treatment before bleomycin administration.

**Key Results:**

Mice undergoing induction of emergency myelopoiesis displayed more severe PH, right ventricular remodelling and pulmonary vascular muscularization compared to controls, without a change in lung fibrosis. This worsening of PH was associated with increased pulmonary myeloid‐derived suppressor cell (MDSC), particularly polymorphonuclear MDSC (PMN‐MDSC). Treatment with αPD‐L1 normalized pulmonary pressures. PD‐L1 expression was likewise found to be elevated on circulating PMN‐MDSC from patients with interstitial lung disease and PH.

**Conclusions and Implications:**

PD‐L1 is a viable therapeutic target in PH, acting through a signalling axis involving MDSC.

**LINKED ARTICLES:**

This article is part of a themed issue on Risk factors, comorbidities, and comedications in cardioprotection. To view the other articles in this section visit http://onlinelibrary.wiley.com/doi/10.1111/bph.v178.1/issuetoc

**Abbreviations**
αPD‐L1: anti‐PD‐L1 antibody
Arg1: arginase 1
DT: diphtheria toxin
ILD: interstitial lung disease
IPF: idiopathic pulmonary fibrosis
MDSC: myeloid‐derived suppressor cell
mDTR: LysM.Cre‐DTR mice
PAH: pulmonary arterial hypertension
PD‐1: programmed cell death protein 1
PD‐L1: programmed death‐ligand 1
PH: pulmonary hypertension
PMN: polymorphonuclear
RV:LV + S: right ventricle to left ventricle plus septal mass ratio
RVSP: right ventricular systolic pressure
Th17: T helper 17 cell
Treg: T regulatory cell

**What is already known**

- Myeloid‐derived suppressor cells contribute to the development of experimental pulmonary hypertension.

**What does this study add**

- Immune checkpoint protein programmed death‐ligand 1 is necessary for pulmonary vascular remodelling.

**What is the clinical significance**

- Immune checkpoint inhibitor therapy may be a viable therapeutic option in patients with pulmonary hypertension.

## 1 INTRODUCTION

Patients with chronic lung disease—such as idiopathic pulmonary fibrosis (IPF)—complicated by pulmonary hypertension (PH; defined as World Health Organization Group 3 PH) are faced with a difficult reality; there is no treatment for their debilitating illness, outside of supplemental oxygen and lung transplant, and they will likely be dead within a year. Therefore, novel targets for disease treatment are urgently required.

Myeloid cells have previously been shown to be implicated in development of PH (Yeager et al., 2012). Proliferation of myeloid cells, specifically those with immunosuppressive capabilities, is known to contribute to aberrant vasculogenesis in several cancers, accounting for increased risk of uncontrolled cellular growth and metastasis (Al Sayed et al., 2019). Moreover, tumour‐associated cytokine signalling in this setting is known to provoke emergency myelopoiesis, an evolutionary response to states of chronic inflammation that is teleologically thought to protect the host against sustained immune activity or autoimmunity (Gabrilovich, 2017). Since innate immune cells display an intrinsically low proliferative capacity, they must be replenished quickly in response to insult such as trauma (Loftus, Mohr, & Moldawer, 2018), infection (Chiba et al., 2018), and—more recently described—chronic inflammatory states of metabolic derangement, such as obesity (Huang, Zhou, et al., 2017). Although the field is expanding (Bryant et al., 2018; Oliveira et al., 2019), there is still relatively little known regarding the mechanistic role of emergency myelopoiesis in the development of pulmonary vascular disease.

Importantly, emergency myelopoiesis as primarily a conserved response to infectious disease replenishes those mature leukocytes lost in combatting illness with immature bone marrow‐derived cells (Chiba et al., 2018). Closely related to neutrophils and monocytes in morphology, myeloid‐derived suppressor cells (MDSCs) are not normally present at high level in steady state, appearing in pathological conditions associated with chronic inflammation or stress. Myeloid‐derived suppressor cells function, in part, by disrupting T cell function through generation of arginase 1 (Arg1) in the micro‐environment depleting availably arginine for normal metabolism, although other immunosuppressive mechanisms have been described (Gabrilovich, 2017). Our group recently demonstrated that emergency myelopoiesis, with an increase in circulating and pulmonary MDSC, can be induced in response to intraperitoneal macrophage apoptosis induced by clodronate liposome injections (Bryant et al., 2018), building upon prior work demonstrating an increase in granulopoiesis, specifically, in response to chronic clodronate liposome administration in a model of cardiac injury (van Amerongen, Harmsen, van Rooijen, Petersen, & van Luyn, 2007). Intriguingly, cell‐specific targeting of myeloid cells themselves—using a LysM.Cre‐DTR, or “mDTR” model—has likewise been shown to elicit increased granulopoiesis with an expansion in circulating Gr‐1^+^ cells after induction of cell death with diphtheria toxin (DT; Goren et al., 2009).

Myeloid cell‐directed immune checkpoint inhibitors applied to existing cancer therapeutic regimens have proven a windfall in the treatment of many malignancies previously felt to be terminal. Likewise, multiple studies have implicated a specific immune checkpoint signalling axis—programmed cell death protein 1 (PD‐1) and the corresponding programmed death‐ligand 1 (PD‐L1)—in the pathogenesis of pulmonary fibrosis (Celada et al., 2018; Geng et al., 2019). Our group has recently published a similar association between severity of PH, as assessed by invasive measurement of mean pulmonary arterial pressure and circulating myeloid cell expression of PD‐L1 in patients with pulmonary arterial hypertension (PAH; World Health Organization Group 1 PH; Bryant et al., 2019). Given these collective findings, we sought to answer the following question: What is the contribution of myeloid cells, specifically myeloid‐derived suppressor cells, PD‐L1 expression to changes in the pulmonary vasculature in response to pulmonary fibrosis? To address this query, we chose to induce widespread, yet controlled, selective cellular apoptosis in a model of pulmonary fibrosis with PH, reporting expression of PD‐L1/PD‐1 as a viable target to promote normal repair of the injured pulmonary circulation.

## 2 METHODS

### 2.1 Animals

LysM.Cre (stock 004781), iDTR (stock 007900) and C57BL/6J (stock 000664) were purchased from the Jackson Laboratory. LysM.Cre and homozygous iDTR were crossed to generate LysM.Cre‐DTR (“mDTR”) mice for experiments as previously described (Goren et al., 2009). All mice (*n* = 5–10 per experimental group) were 10–12 weeks of age at the study onset, included equal numbers of both males and females and ranged in weight from 20 to 30 g. All animal studies were approved by the University of Florida Institutional Animal Care and Use Committee (IACUC; Protocol 08702). Animal studies are reported in compliance with the ARRIVE guidelines (Kilkenny, Browne, Cuthill, Emerson, & Altman, 2010) and with the recommendations made by the *British Journal of Pharmacology.*

### 2.2 Statistical analysis

Studies were designed to generate exposure and treatment groups of equal size, randomized to condition, with blinded analysis. Statistical analysis was undertaken only for studies where each group size was at least *n* = 5. Group sizes represent the number of independent values, with statistical analysis done using these values, not treating technical replicates as independent values. Where indicated, variable relative units were determined using fold matched control values, and not fold mean of control values, with data normalization of values to control group values. We determined a priori that 10 bleomycin‐treated mice are needed for each harvest. This number is based on prior experience, anticipated animal mortality due to bleomycin toxicity and power analysis that demonstrates that 10 mice are needed in each arm to detect a difference of 20% expression with 80% power at *α* = .05 assuming SD of 15%. No outliers were excluded in data analysis and presentation. Statistical analyses were performed with GraphPad InStat (GraphPad Prism, RRID:SCR_002798). All graphing and statistical analyses were carried out using GraphPad Prism (GraphPad Prism, RRID:SCR_002798). All animal data are presented as mean ± SEM. The Student's *t*‐test was used for single comparisons and two‐way ANOVA was used for multiple comparisons. Post hoc tests were conducted only if ANOVA achieved the necessary level of statistical significance, in absence of variance homogeneity indicated nonparametric values. Human data are presented as median ± interquartile range. The Mann–Whitney *U* test or the Kruskal–Wallis rank‐sum test was used for non‐normal data, with Dunn's multiple comparison test. *P* < .05 was considered significant. The data and statistical analysis comply with the recommendations of the *British Journal of Pharmacology* on experimental design and analysis in pharmacology (Curtis et al., 2018).

### 2.3 Bleomycin‐induced pulmonary fibrosis and PH

Mice underwent intraperitoneal injection with 0.018 U·g^−1^ bleomycin (Thermo Fisher Scientific) or vehicle (PBS) twice weekly for 4 weeks (Pi et al., 2018). For diphtheria toxin studies, mice were injected intraperitoneally for three consecutive days with 100 ng of diphtheria toxin (Sigma Aldrich) before initiation of bleomycin protocol, with PBS injection used as control (Goren et al., 2009), and twice weekly thereafter. Euthanasia and data collection are performed 5 days after final injection of animal with vehicle or experimental agent (diphtheria toxin and bleomycin), Day 33 of the intraperitoneal bleomycin injection protocol. Anti‐PD‐L1 (500 μg, intraperitoneally) and rat IgG2b, both purchased from BioXcell, were administered on Day 0 and then once weekly for three additional doses (Celada et al., 2018) throughout bleomycin exposure.

### 2.4 Flow cytometry, antibodies, PCR, western blot and multiplex array

Flow cytometry analyses were performed on a BD LSR II or on FACSCalibur upgraded at three lasers and eight colours (Cytek). Cell populations were identified using sequential gating strategy characterized within body of manuscript (excluding debris and doublets). Fluorescence minus one and isotype controls were used when necessary. The expression of markers is presented as median fluorescence intensity. Data were analysed using FlowJo software (FlowJo, RRID:SCR_008520). A comprehensive list of all antibodies used in detailed experiments is provided in Table S1 and as detailed previously (Bryant et al., 2018; Misharin, Morales‐Nebreda, Mutlu, Budinger, & Perlman, 2013). Reverse transcriptase PCR was performed on cDNA (Qiagen). Indicated are the sequences of 5′ and 3′ primers used for each of the tested targets: mus 18S, forward 5′‐GTAACCCGTTGAACCCCATT‐3′, reverse 5′‐CCATCCAATCGGTAGTAGCG‐3′; mus fibronectin, forward 5′‐TACAATGTGGGACCCTTGGC‐3′, reverse 5′‐TGGTTCCCTTTCACAGCCAC‐3′; and mus type 1 collagen α1, forward 5′‐GAAGCACGTCTGGTTTGGA‐3′, reverse 5′‐ACTCGAACGGGAATCCATC‐3′. Western blot was performed using the primary antibodies (5% nonfat milk): anti‐Collagen1a1 (Novus Biologicals Cat# NB600‐408, Epitope Collagen I from human and bovine placenta. Uniprot ID P02452; diluted 1:2,500), anti‐Fibronectin (Novus Biologicals Cat# NBP1‐91258, Epitope synthetic peptide made towards the C‐terminal region of the human Fibronectin protein (within residues 2,250–2,300). [Swiss‐Prot: P02751]; diluted 1:2,500), and anti‐β‐actin (Cell Signaling Technology Cat# 4967, Epitope Swiss‐Prot Acc.: P60709; diluted 1:5,000), overnight at 4°C. The membranes were washed three times with PBST and then incubated with anti‐rabbit (Jackson ImmunoResearch Labs Cat# 111‐035‐144; diluted 1:5,000) HRP‐conjugated antibodies for 2 hr at room temperature. The immune complexes were visualized by the ECL chemiluminescence method (Ultra TMA‐6, Lumigen). A multiplex array (Millipore) was used to detect and quantify mouse cytokine/chemokines in whole lung homogenate using previously described techniques (Bryant et al., 2018). Data were acquired using a Luminex®200™ and analysed using Milliplex Analyst Software (VigeneTech Inc). The Immuno‐related procedure reporting complies with the editorial guidelines on immunoblotting and immunohistochemistry (Alexander et al., 2018).

### 2.5 Pulmonary haemodynamic and histologic assessments

Upon completion of experimental protocols, intact mice underwent invasive closed‐chest measurement of right ventricular systolic pressure (RVSP). In brief, a Millar 1.4 French pressure‐volume microtip catheter transducer (SPR‐839; Millar Instruments) connected to a PowerLab/8s (ADInstruments) was inserted through a right internal jugular vein incision and threaded down into the right ventricle. RVSP (mmHg) recordings were collected using Chart 5 (ADInstruments). Bronchoalveolar lavage was performed where indicated as previously described (Degryse et al., 2010), with standard BCA later performed for evaluation of protein concentration. Upon completion of the measurements, the heart was excised with removal of the atria along with the right (RV) and left ventricles (LV) plus septum. They were isolated for measurement of the RV:LV + S as previously described (Hemnes et al., 2014). Formalin‐fixed, paraffin‐embedded, lung was then assessed histologic fibrosis score (Tanjore et al., 2013). Lung histology was additionally stained for α‐smooth muscle actin (Abcam, rabbit polyclonal, Cat# ab5694; Epitope a synthetic peptide corresponding to N‐terminus of actin from human smooth muscle; diluted 1:750 in antibody diluent reagent solution [Life Technologies], not reused; blocking reagent Background Sniper [Biocare]) using mouse gastrointestinal tract as a positive control and assessed, which was blinde for muscularized pulmonary vessel count (Bryant et al., 2015). Images were obtained using a Keyence BZ‐X microscope, with analysis performed using BZ‐X analyser software (Keyence).

### 2.6 T‐cell proliferation assay

T‐cell proliferation/suppression assays were performed as previously described (Highfill et al., 2014). In brief, wild‐type C57BL/6 T cells were isolated with CD3 monoclonal antibody‐coated magnetic beads (Miltenyi Biotec) and stained with Cell Trace Violet. These cells were then stimulated with anti‐CD3/CD28 mouse beads (Thermo Fisher). Murine myeloid‐derived suppressor cells were isolated from spleens of mDTR mice by Myeloid‐Derived Suppressor Cell Isolation Kit (Miltenyi Biotec) after lysis of red blood cells according to the manufacturer's instructions, as previously described (Oliveira et al., 2019). The ratio between splenic myeloid‐derived suppressor cells and T cells was tested at 4:1, 2:1 and 1:1. Cells were incubated for 5 days, with proliferation assessed as Cell Trace Violet dilution by flow cytometry and per cent proliferation calculated for each group.

### 2.7 Study approval

Human sample use was approved by the Institutional Review Board of Rhode Island Hospital. Subjects were enrolled from the Rhode Island Hospital Pulmonary Hypertension Center as part of a local registry and biorepository (Baird et al., 2018), which captures all patients referred for PH evaluation. Subjects were included if they were clinically phenotyped as pulmonary fibrosis with no PH or pulmonary fibrosis with PH by standard haemodynamic and/or echocardiographic criteria, as previously described (Ventetuolo et al., 2016). Definition of PH in patients was determined by 2013 guidelines, mean pulmonary arterial pressure greater than or equal to 25 mmHg at rest (Hoeper et al., 2013). Detailed demographic data of patient cohorts can be found in Table S2.

### 2.8 Nomenclature of targets and ligands

Key protein targets and ligands in this article are hyperlinked to corresponding entries in http://www.guidetopharmacology.org, the common portal for data from the IUPHAR/BPS Guide to PHARMACOLOGY (Harding et al., 2018), and are permanently archived in the Concise Guide to PHARMACOLOGY 2019/20 (Alexander et al., 2019).

## 3 RESULTS

### 3.1 Emergency myelopoiesis contributes to pulmonary vascular remodelling, and PH, with no alteration in pulmonary fibrosis

**FIGURE 1:**
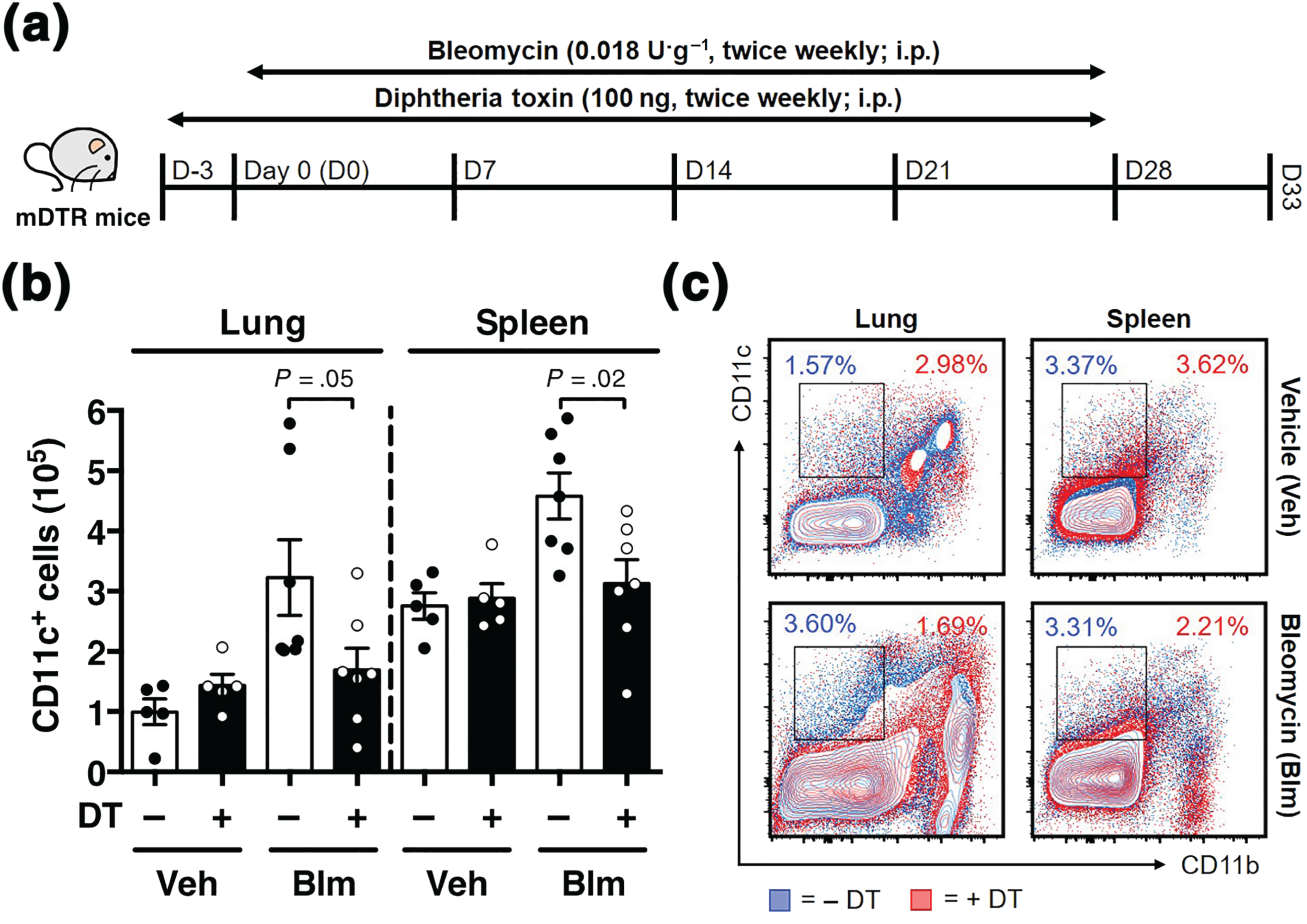
Diphtheria toxin (DT) administration to LysM.Cre‐DTR (“mDTR”) mice results in a decrease in CD11c^+^ cells in the lung and spleen of mice given bleomycin. (a) Schematic demonstrating the protocol for stimulation of myelopoiesis, with harvest occurring at Day 33 (D33) after initiation of bleomycin protocol. (b) Injection (100 ng, intraperitoneal, twice weekly) of DT resulted in an absolute and relative (c: representative flow cytometric plots) decrease in the amount of CD11c^+^ cells within the lung and spleen of vehicle (Veh)‐ or bleomycin (Blm)‐stimulated mDTR mice. *n* = 5 mice per vehicle group, and 7 mice per bleomycin group. All data are presented as mean ± SEM. *P* < .05 was considered significant

In order to test our hypothesis that induction of emergency myelopoiesis using the mDTR model would result in an increase in myeloid‐derived cells in a model of pulmonary vascular disease, we stimulated bleomycin‐associated pulmonary fibrosis and PH in mDTR mice administered concurrently either vehicle or diphtheria toxin, in order to induce emergency myelopoiesis (Figure 1a). First, however, we wanted to confirm that, in a population of CD11b^lo^CD11c^+^ cells within the lung and spleen, inclusive of macrophage subsets expected to undergo apoptosis, we could achieve an expected reduction in cell numbers upon diphtheria toxin administration. We were able to successfully demonstrate such a reduction in absolute cell numbers upon completion of the full diphtheria toxin injection protocol, but only significantly in mice co‐stimulated by the bleomycin administration (Figure 1b,c).

**FIGURE 2:**
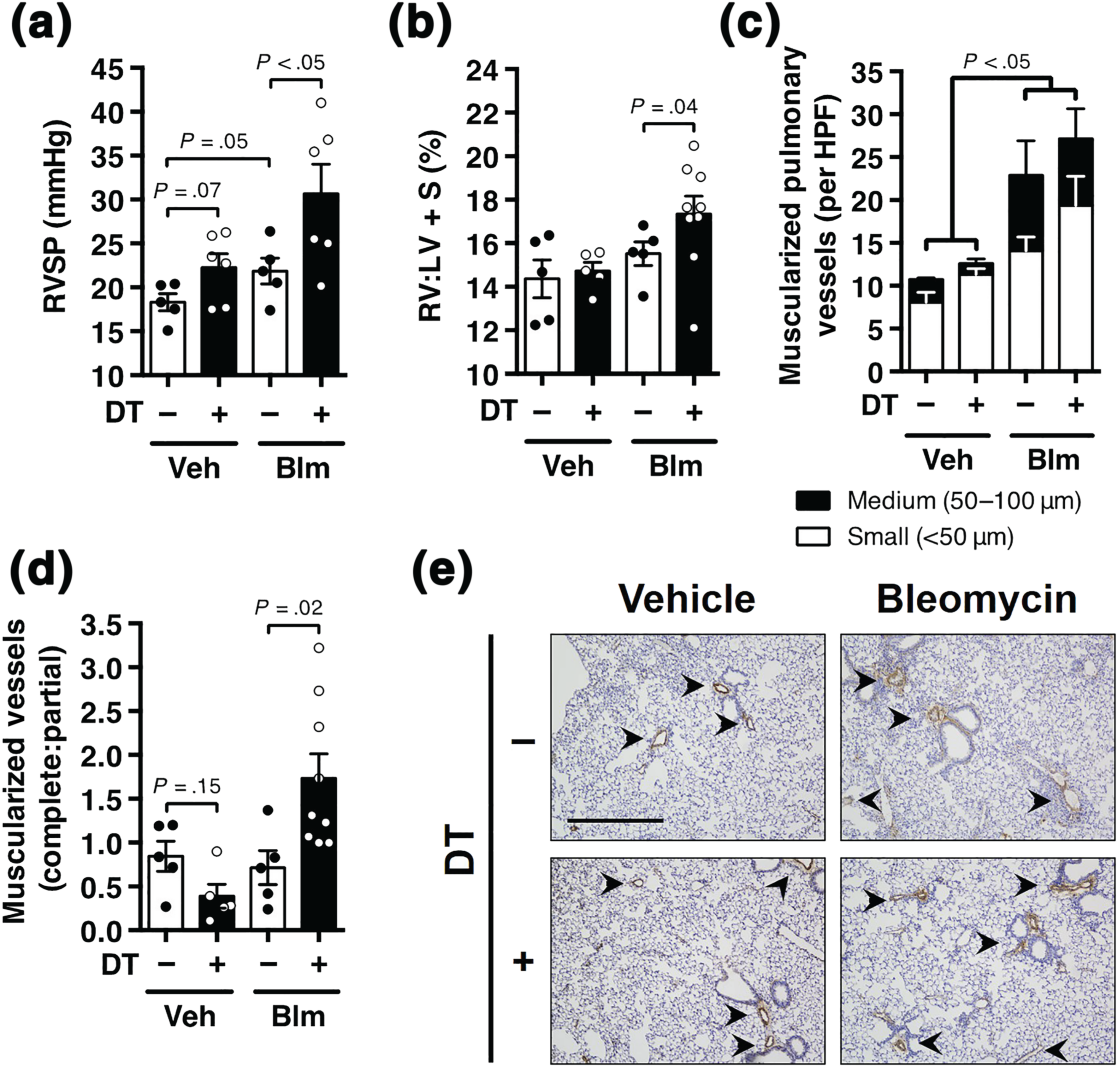
Diphtheria toxin (DT) administration to LysM.Cre‐DTR (“mDTR”) mice given bleomycin results in an increase in pulmonary hypertension. (a) Right ventricular systolic pressure (RVSP; mmHg) as measured invasively in mDTR mice given vehicle (Veh) or bleomycin (Blm) with or without DT. (b) Right ventricle to left ventricle plus septal mass ratio (RV:LV + S; %) between groups. (c) Absolute muscularized vessel counts per high‐powered field (HPF), and (d) ratio of complete‐to‐partially muscularized vessels, as assessed by (e) α‐smooth muscle actin (α‐sma; brown, arrowheads) immunohistochemical (IHC) stain of sectioned lungs from treatment groups as indicated. Scale bar 500 μm. *n* = 5 mice per vehicle group and 9 mice per bleomycin group. All data are presented as mean ± SEM. *P* < .05 was considered significant

**FIGURE 3:**
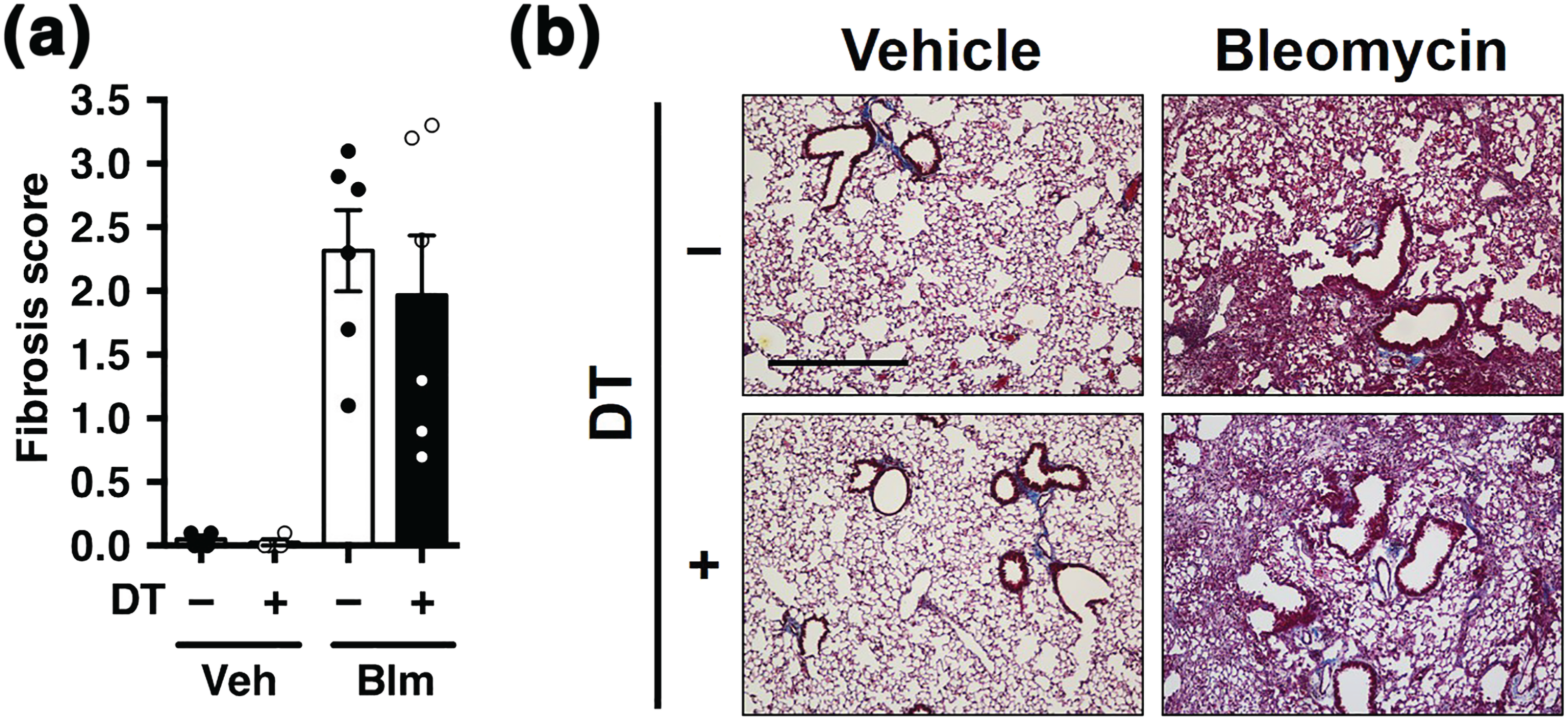
Diphtheria toxin (DT) administration to LysM.Cre‐DTR (“mDTR”) mice given bleomycin results in no change in pulmonary fibrosis. (a) Fibrosis score quantified between mDTR mice groups administered vehicle (Veh) or bleomycin (Blm), with or without DT. (b) Masson's trichrome stain (MTC) of sectioned lungs from treatment groups. Scale bar 500 μm. *n* = 5 mice per vehicle group, and 6 mice per bleomycin group. All data are presented as mean ± SEM

Next, we measured RVSP (mmHg) in mDTR mice given bleomycin or vehicle. Consistent with our prior data using the clodronate liposome model (Bryant et al., 2018; Pi et al., 2018), mDTR mice given bleomycin and diphtheria toxin displayed significantly higher RVSP than control mice (Figure 2a), with an expected increase in right ventricular remodelling, as assessed by the right ventricle to left ventricle plus septal mass ratio (RV:LV + S; %; Figure 2b). Additionally, although there was no significant difference between bleomycin‐treated groups in absolute small‐ and medium‐sized pulmonary vessel muscularization (Figure 2c), there was a significant increase in the ratio of fully to partially muscularized vessels (Figure 2d), assessed by α‐smooth muscle actin staining (Figure 2e). Importantly, there was no significant difference in pulmonary fibrosis, as assessed by either the modified Ashcroft score (Figure 3a) or reverse transcriptase PCR analysis of fibrotic markers collagen 1 and fibronectin (Figure S1A,B), between bleomycin‐treated groups (Figure 3b). Additionally, we found no significant difference between bleomycin‐treated groups in bronchoalveolar lavage fluid protein concentration (Figure S1C), implying no differences in epithelial barrier integrity contributing to our phenotype. We also found no difference in apoptotic cell count between bleomycin‐treated groups (Figure S1D,E) at the conclusion of our 33‐day protocol, suggesting that LysM Cre‐recombinase off‐target cellular death—in lung epithelial cells, for example (McCubbrey, Allison, Lee‐Sherick, Jakubzick, & Janssen, 2017)—did not play a significant role in our findings. However, it is relevant to note that diphtheria toxin alone induces a significant increase in apoptosis in the absence of bleomycin. We therefore cannot rule out the possibility that early stage disease could be significantly impacted by epithelial cell loss in these mice, with changes in the described measurements being obscured as disease progresses. Given these caveats, we conclude that relatively selective cell depletion of myeloid cells, using the mDTR mouse model, results in worsened PH that cannot be attributed to major differences in lung fibrosis and vessel dropout.

### 3.2 Emergency myelopoiesis stimulates myeloid‐derived suppressor cell movement to the lung in setting of PH

**FIGURE 4:**
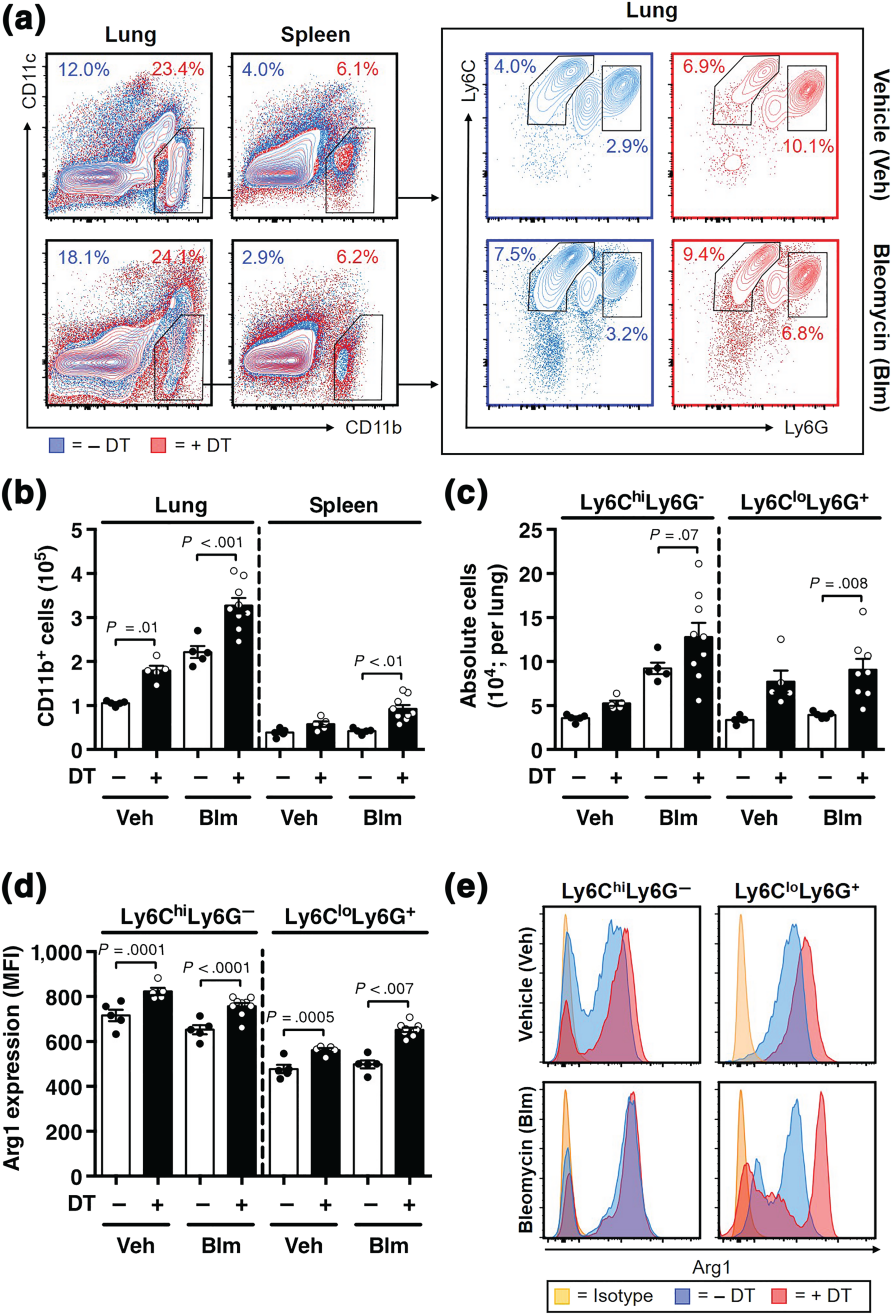
LysM.Cre‐DTR (“mDTR”) mice given diphtheria toxin (DT) and bleomycin display an increase in pulmonary myeloid‐derived cells, characterized by an immunosuppressive signature. (a) Representative flow cytometric plots displaying relative differences in CD11b^+^ cell population within lung and spleen, including pulmonary Ly6C^hi^Ly6G^−^ and Ly6C^lo^Ly6G^+^ cells, in mDTR mice with and without DT treatment, and vehicle or bleomycin. (b, c) Absolute cell counts for treatment groups. (d, e) Arginase 1 (Arg1) expression by mean fluorescence intensity (MFI) in treated groups of pulmonary Ly6C^hi^Ly6G^−^ and Ly6C^lo^Ly6G^+^ cells, with representative histogram. *n* = 5 mice per vehicle group and 9 mice per bleomycin group. All data are presented as mean ± SEM. *P* < .05 was considered significant

In order to assess and characterize evidence of emergency myelopoiesis in our model, we further examined lung and spleen from experimental mice for confirmation of increased CD11b^+^ cell subsets. Using flow cytometry, we found that mice given diphtheria toxin plus vehicle or bleomycin displayed a substantial increase in primarily lung CD11b^+^ absolute cell counts (Figure 4a,b), with a simultaneous rise in the number of pulmonary Ly6C^hi^Ly6G^−^ (“monocytes”) and Ly6C^lo^Ly6G^+^ (“neutrophils”) cells, although only significantly increased in the latter population (Figure 4c). Consistent with these findings, when tissue homogenate was analysed using a cytokine/chemokine array, expected granulocytic growth factors were elevated (G‐CSF and Eotaxin), yet—surprisingly—other markers of inflammation were unexpectedly decreased in lungs from mice given diphtheria toxin compared to bleomycin‐treated controls (IL‐1β, IL‐2, MIP‐2, and RANTES; Figure S2A–F).

Our group has previously shown that such an immunosuppressive signature is associated with Arg1 up‐regulation in putatively neutrophil‐like myeloid‐derived cells, influenced largely by emergency myelopoiesis (Bryant et al., 2018). Therefore, we analysed levels of Arg1 in the identified monocyte and neutrophil subpopulations, determining that protein expression was elevated in those cells from diphtheria toxin‐treated mice and both vehicle‐ and bleomycin‐treated groups compared to vehicle controls (Figure 4d,e).

**FIGURE 5:**
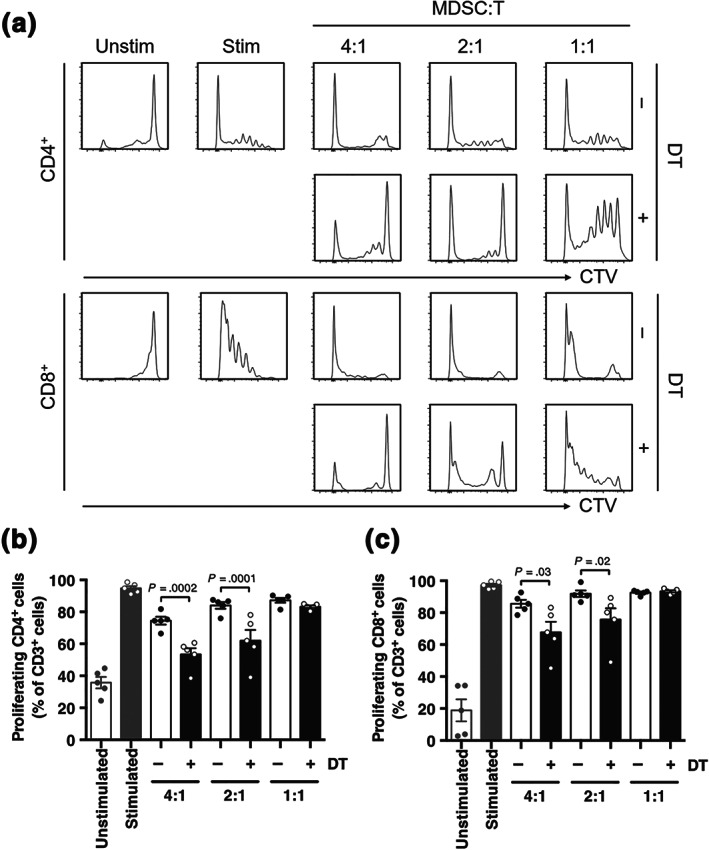
Myeloid cells isolated from LysM.Cre‐DTR (“mDTR”) mice given diphtheria toxin (DT) suppress T cell proliferation, characteristic of myeloid‐derived suppressor cells (MDSCs). (a–c) Representative histograms and proliferation index (CD4^+^ and CD8^+^ cells, as % of T cells) after MDSC purified from spleens of mDTR mice treated with and without DT were cultured with T cells labelled with Cell Trace Violet (CTV) and stimulated with anti‐CD3/CD28 antibodies at listed ratios. *n* = 5 mice per group. All data are presented as mean ± SEM. *P* < .05 was considered significant

We therefore wanted to next determine if myeloid‐derived suppressor cells presence in our model could possibly account for the aforementioned immunosuppressive profile. Isolating Gr‐1^+^ cells from either vehicle‐ or diphtheria toxin‐treated mDTR mice, we co‐cultured these “MDSC myeloid‐derived suppressor cells” with T cells in different ratios, examining for lymphocyte proliferation upon antibody‐mediated stimulation (Figure 5a). We found that both CD4^+^ (Figure 5b) and CD8^+^ (Figure 5c) T cells were suppressed in response to isolated myeloid‐derived cells from diphtheria toxin‐exposed animals, compared to stimulated controls. Therefore, we determined that the inhibition of proliferating CD4^+^ and CD8^+^ cells tracked with increasing amount of co‐culture with these now fully defined myeloid‐derived suppressor cells. Given these findings, the previously referred to “neutrophils” (Figure 4b,c) were thereafter to be referred to as polymorphonuclear myeloid‐derived suppressor celsl (PMN‐MDSC), recognizing that these cells were responsible for the displayed increase in lung CD11b^+^Ly6C^lo^Ly6G^+^ cells. From these data, we concluded that mDTR mice treated with diphtheria toxin and bleomycin undergo emergency myelopoiesis, ultimately yielding an increase in lung myeloid‐derived suppressor cells, specifically PMN‐MDSC, associated with development of PH.

### 3.3 Immune cell expression of PD‐1/PD‐L1 is enhanced in PH secondary to pulmonary fibrosis

**FIGURE 6:**
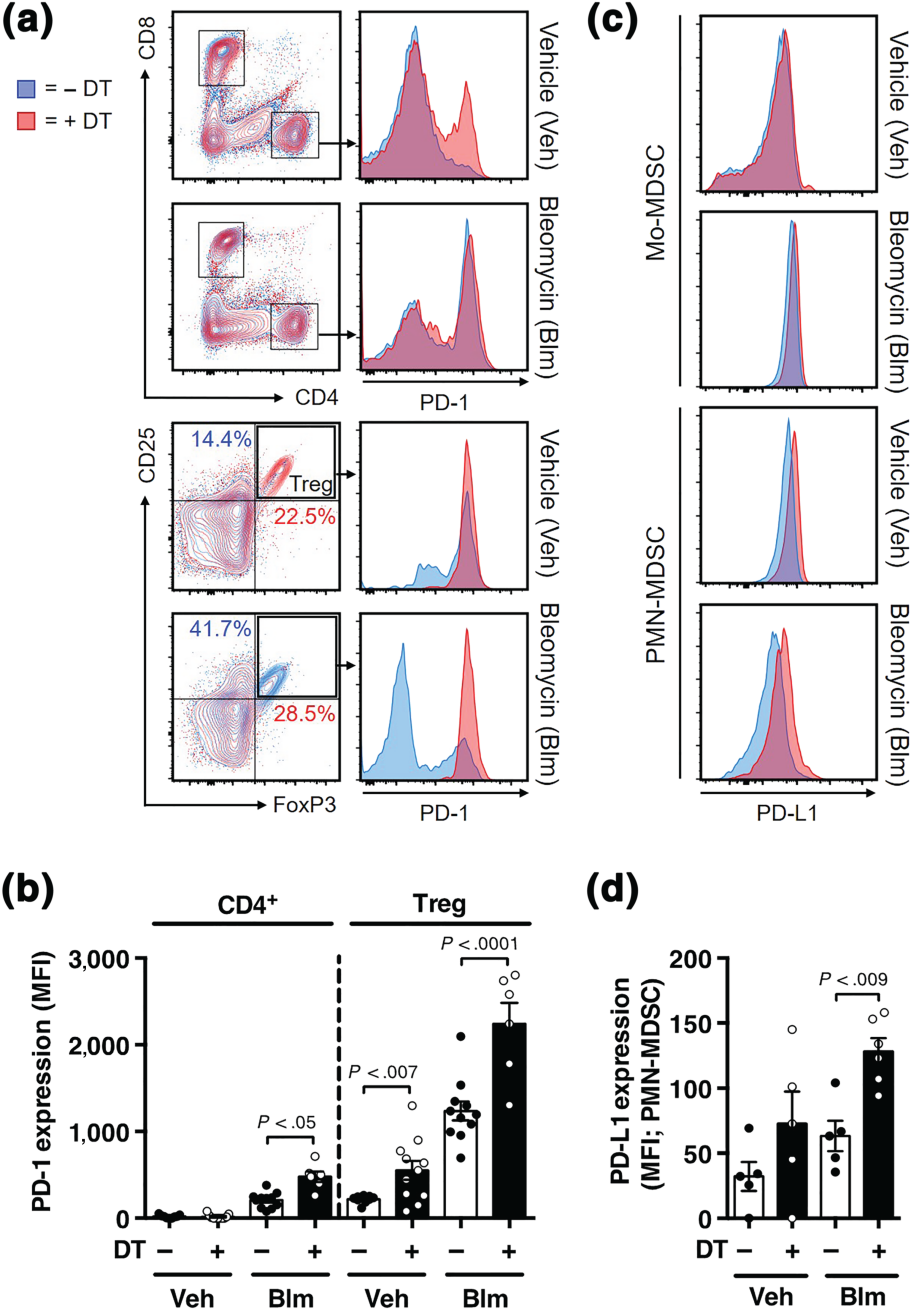
LysM.Cre‐DTR (“mDTR”) mice given diphtheria toxin (DT) have increased pulmonary expression of PD‐1 in T cells and PD‐L1 in myeloid‐derived suppressor cells (MDSC). (a) Representative flow plots and histograms detailing CD4^+^ and Treg cell—with relative abundance per CD4^+^ cells (%)—expression of PD‐1 by mean fluorescence intensity (MFI) upon treatment of mDTR mice with or without DT in combination with vehicle (Veh) or bleomycin (Blm). (b) Quantification of expression data by MFI in subpanel (a). (c) Representative histograms of PD‐L1 expression in MDSC subpopulations (monocytic, Mo‐, and polymorphonuclear, PMN‐MDSC) within exposed treatment groups. (d) Quantification of expression data by MFI in subpanel (b). *n* = 11 mice per group, except in PD‐L1 expression where *n* = 6 mice per group. All data are presented as mean ± SEM. *P* < .05 was considered significant

Myeloid‐derived suppressor cells presence is known to be associated with T cell exhaustion and senescence via PD‐1 up‐regulation on effector T cells, establishing the rationale for PD‐1/PD‐L1 signalling blockade as an effective immune therapy for many cancers (Huang, Francois, McGray, Miliotto, & Odunsi, 2017). Thus, we determined the level of PD‐1 expression on T cell subpopulations in our model of PH. We found significant differences in CD4^+^ and CD4^+^CD25^+^FoxP3^+^ (Treg) cell expression of PD‐1 (Figure 6a,b), the latter group displaying elevated PD‐1 in both vehicle‐ and bleomycin‐exposed diphtheria toxin‐treated mice lungs compared to controls.

Vascular injury is known to lead to increased PD‐L1 expression by circulating myeloid‐derived suppressor cells, with anti‐PD‐L1 therapy leading to subsequent improved T cell activation (Noman et al., 2014). In particular, myeloid‐derived suppressor cells facilitate an increase in exhaustive Treg in chronic inflammatory states, associated with increased PD‐L1 expression (Lee et al., 2016). We found that while there was no increase in PD‐L1 expression by the monocytic myeloid‐derived suppressor cells (Mo‐MDSC; defined as live singlets with surface markers CD45^+^CD11b^+^CD11c^−^Ly6C^hi^Ly6G^−^) subgroup, the neutrophilic myeloid‐derived suppressor cells (PMN‐MDSC; live singlets with surface markers CD45^+^CD11b^+^CD11c^−^Ly6C^lo^Ly6G^+^; Bronte et al., 2016) population displayed a significant elevation in PD‐L1 in the lungs of mice treated with both diphtheria toxin and bleomycin, compared to control animals (Figure 6c,d). These data support the conclusion that T cell PD‐1—and PMN‐MDSC PD‐L1—expression is associated with development of worsening PH in the bleomycin‐injury model.

### 3.4 Anti‐PD‐L1 prevents development of PH secondary to pulmonary fibrosis through alteration in the effector T cell response

**FIGURE 7:**
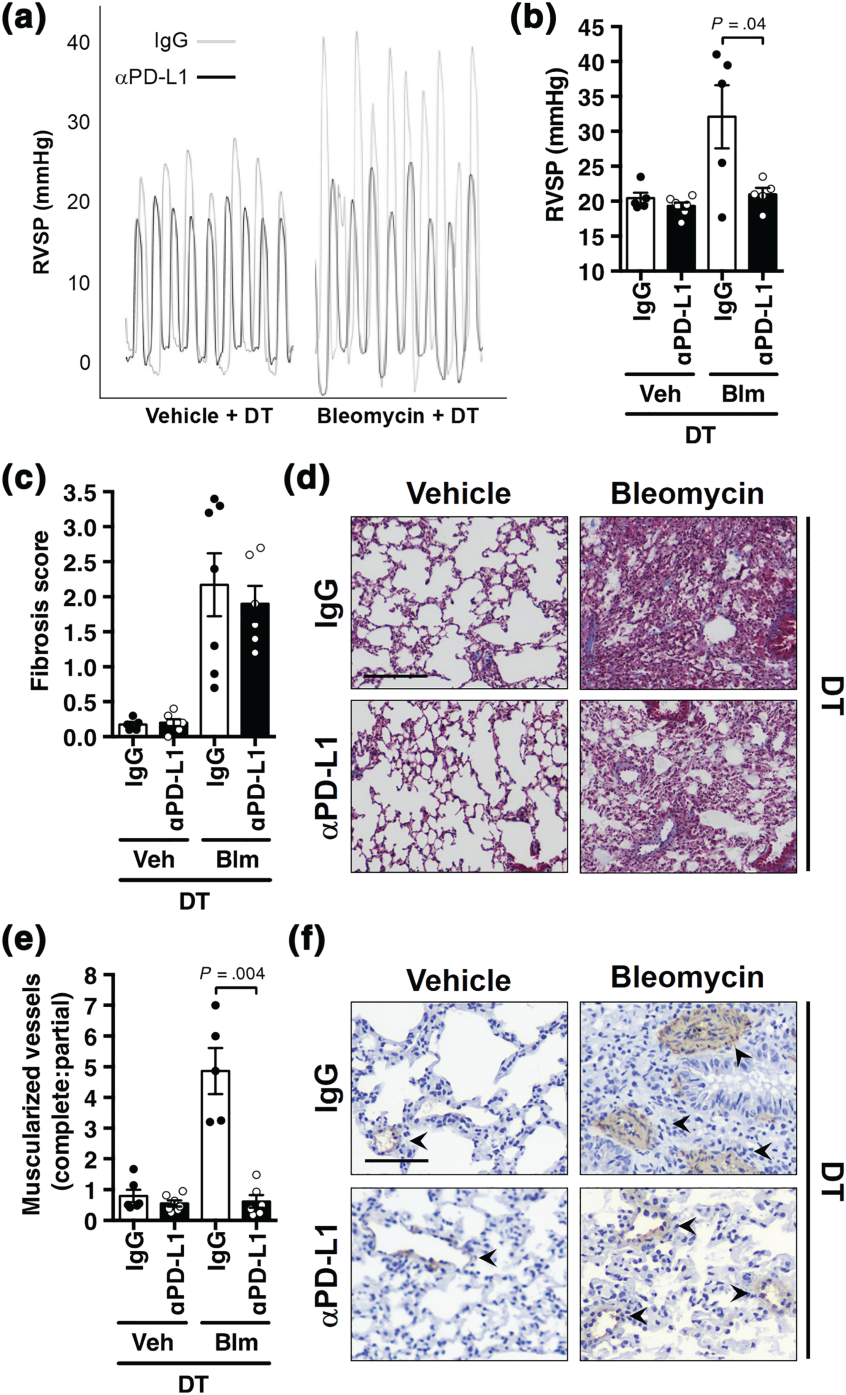
Anti‐PD‐L1 treatment protects against development of pulmonary hypertension, but not pulmonary fibrosis, after induction of emergency myelopoiesis in the bleomycin (Blm) model. (a) Illustrative right ventricular systolic pressure (RVSP)‐time tracing in diphtheria toxin (DT)‐treated LysM.Cre‐DTR (“mDTR”) mice exposed to vehicle (Veh) or bleomycin (Blm), treated with anti‐PD‐L1 antibody or IgG control. (b) RVSP (mmHg) and (c) fibrosis score in described groups. (d) Masson's trichrome stain (MTC) of lung sections from treatment groups. (e) Complete‐to‐partially muscularized pulmonary vessel ratio, assessed by (f) α‐smooth muscle actin (α‐sma; brown, arrowheads) stain of lung sections. Scale bars 100 μm. *n* = 5 mice per vehicle group, and 7 mice per bleomycin group. All data are presented as mean ± SEM. *P* < .05 was considered significant

Next, in order to test PD‐L1 as a viable therapeutic target for PH prevention, we treated mDTR mice—all receiving diphtheria toxin—with either anti‐PD‐L1 antibody (αPD‐L1) or immunoglobulin control (IgG) using the bleomycin model. Mice given αPD‐L1 preventively displayed near normal RVSP upon bleomycin treatment, compared to immunoglobulin‐treated controls (Figure 7a,b). Though there was no change in the degree of fibrosis (Figures 7c,d and S3A) or apoptotic cell count (Figure S3B,C) between bleomycin‐treated groups, there was a corresponding decrease in ratio of fully to partially muscularized pulmonary vessels compared to vehicle control‐treated animal values (Figure 7e,f). These data highlight the absence of relationship between degree of fibrosis and development of PH, a similar phenomenon to that seen in patients with IPF and PH.

**FIGURE 8:**
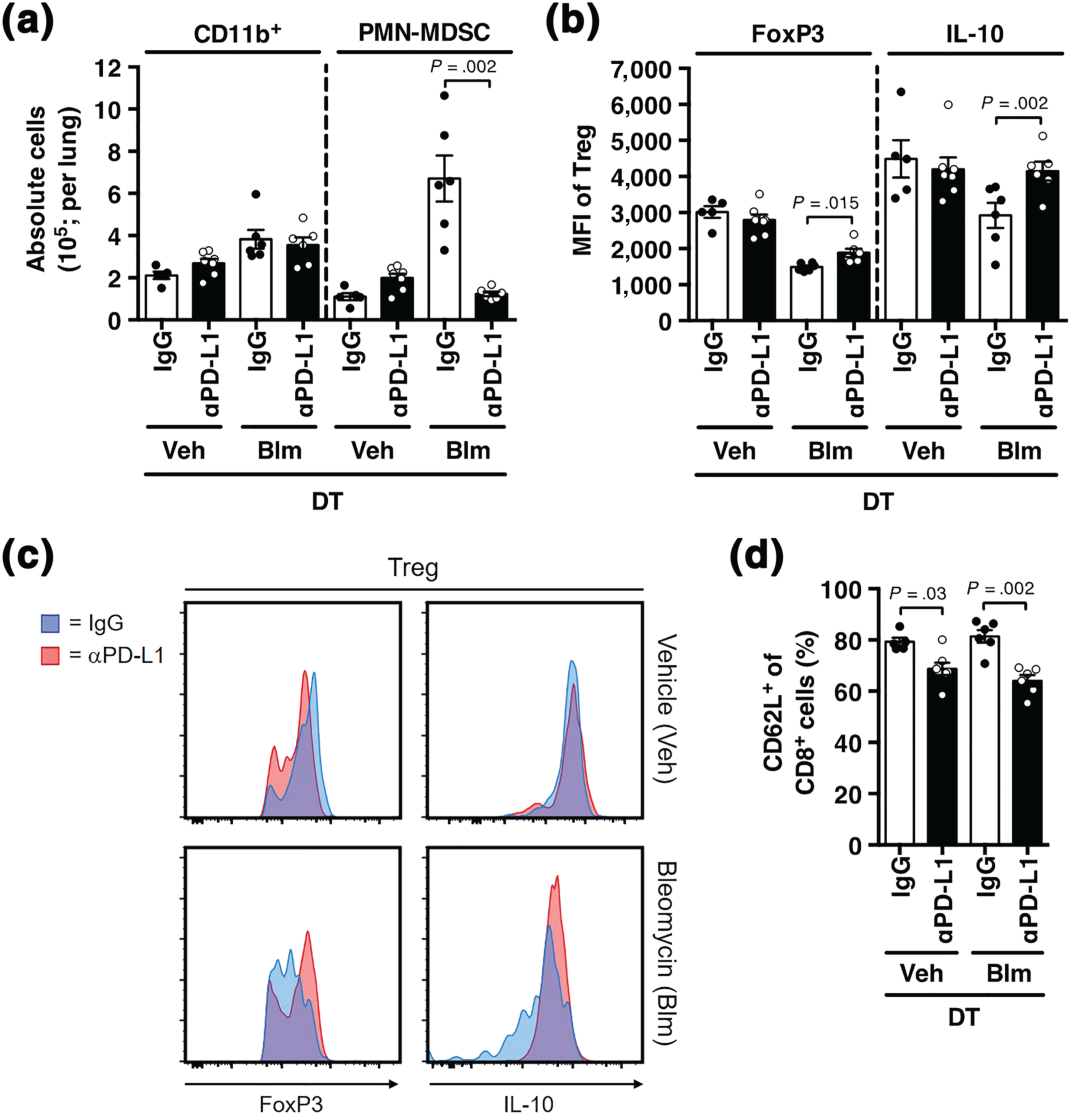
Anti‐PD‐L1 treatment results in a decrease in myeloid‐derived suppressor cells (MDSC) within the lung, and improvement in markers of Treg exhaustion upon induction of emergency myelopoiesis in the bleomycin model. (a) Absolute CD11b^+^ and polymorphonuclear MDSC (PMN‐MDSC) counts after treatment with either IgG control or anti‐PD‐L1 antibody in vehicle (Veh)‐ or bleomycin (Blm)‐exposed LysM.Cre‐DTR (“mDTR”) mice given diphtheria toxin (DT). (b) Expression of FoxP3 and IL‐10 in pulmonary Treg cells of described groups, with (c) representative histograms. (d) Percentage of CD62L^+^ cells (of CD8^+^ T cells) in treatment groups. *n* = 6 mice per group. All data are presented as mean ± SEM. *P* < .05 was considered significant

To explore a potential mechanism of αPD‐L1 action, we explored the effect of treatment on myeloid‐derived suppressor cells and T lymphocyte subgroups. First, we showed that despite a lack of difference in absolute CD11b^+^ cell count between antibody‐treated groups given bleomycin, there was a significant decrease in the number of lung PMN‐MDSC with αPD‐L1 injections (Figure 8a). Interestingly, associated with this observed decrease was an increase in FoxP3 and IL‐10 expression by pulmonary Treg in mice given αPD‐L1 (Figure 8b,c). We also noted a decrease in CD62L^+^ cells—as a percentage of CD8^+^ effector T cells (Figure 8d)—consistent with increased Treg capacity. Taken together, we conclude that the PD‐1/PD‐L1 signalling axis is a viable target for prevention of PH, potentially acting through alterations in a complex regulatory network of pulmonary PMN‐MDSCs and T lymphocytes.

### 3.5 Patients with interstitial lung disease complicated by PH display an increase in PD‐L1 expression by circulating myeloid cells

**FIGURE 9:**
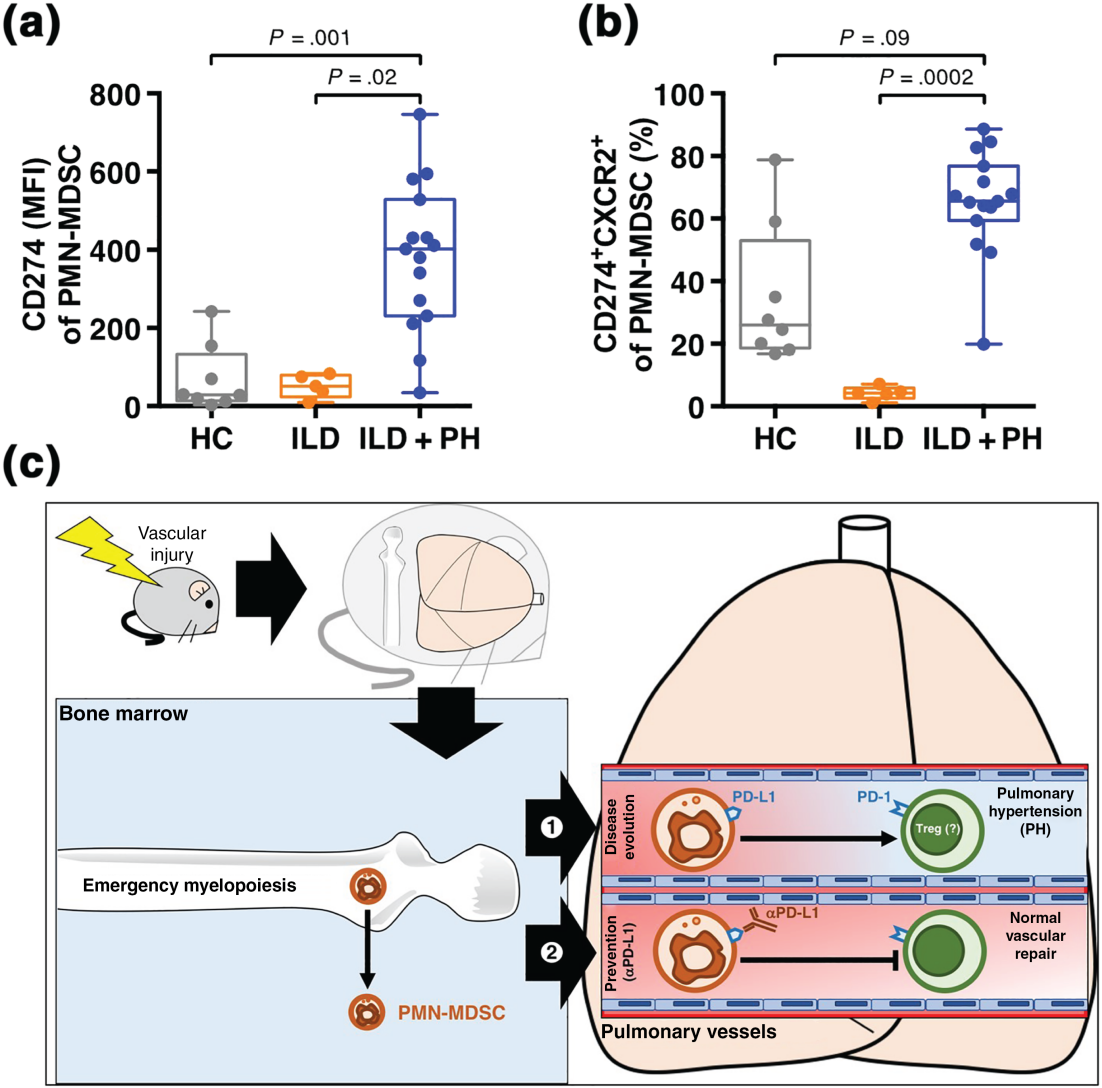
Patients with interstitial lung disease (ILD) and pulmonary hypertension (PH) have increased expression of PD‐L1 (CD274) on MDSC. (a) Expression of CD274 (PD‐L1) in mean fluorescence intensity (MFI) by CD11b^+^CD33^+^HLA‐DR^−^CD14^−^CD15^+^ cells (PMN‐MDSC) in HC (*n* = 8) and ILD with (*n* = 15), and without PH (*n* = 5) patient peripheral blood samples. (b) Percentage of CD274^+^CXCR2^+^ cells (of PMN‐MDSC) between HC and ILD groups. (c) Summary figure. All data are presented as median ± interquartile range. *P* < .05 was considered significant

Given the pre‐clinical relevance these studies suggest in conceivable treatment of patients with Group 3 PH, we next sought to investigate differences in myeloid cell expression of PD‐L1 (CD274) in peripheral blood samples from healthy controls and patients with interstitial lung disease with (ILD + PH) and PH without interstitial lung disease. Using a previously described classification schema (Bronte et al., 2016), we quantified CD274 expression on live singlet CD33^+^CD11b^+^CD14^−^CD15^+^ cells (PMN‐MDSC, human) and found that cells from patients with ILD + PH displayed higher levels of the checkpoint protein, compared to both controls and patients with only interstitial lung disease (Figure 9a). These data are consistent with our own published work documenting an increase in CD274 expression by myeloid‐derived suppressor cells in a separate cohort of patients with PAH (Bryant et al., 2019).

Finally, chemokine receptor CXCR2 has been previously shown to act synergistically with PD‐L1 in oncologic models of myeloid‐derived suppressor cells—specifically PMN‐MDSC—recruitment and activation (Highfill et al., 2014). Moreover, CXCR2^+^ PMN‐MDSCs, acting directly through PD‐1/PD‐L1, induce CD4+ T cell exhaustion (Zhu et al., 2017). We therefore hypothesized that patients with ILD + PH would have more CD274^+^CXCR2^+^ PMN‐MDSC, consistent with these prior observations. While the percentage of these dual‐positive cells was not significantly increased compared to control subjects, there were markedly more CD274^+^CXCR2^+^ PMN‐MDSC in the circulation of patients with ILD + PH, compared to those with just ILD (Figure 9b). Cumulatively, these data indicate the applicability of the murine model (Figure 9c) to the human pathological condition and represent a viable future therapeutic target for patients with disease.

## 4 DISCUSSION

Group 3 pulmonary hypertensive patients have no disease‐specific therapy to date, despite having a worse prognosis than demographically similar populations with advanced malignancy. As a novel area of study in the field, it is known that dysregulated myelopoiesis in response to chronic inflammatory injury can lead to an exhausted cellular phenotype with long‐term immunosuppression, vascular injury (Boettcher et al., 2014), decreased functional status (Loftus et al., 2018) and uncontrolled cellular proliferation (Strauss, 2015). Related granulopoiesis and subsequent neutrophilia is known to be an independent risk factor for early death in idiopathic pulmonary fibrosis (IPF; Kinder et al., 2008). Likewise granulocytes have been implicated as pathogenic in a number of pulmonary vascular diseases (Taylor, Dirir, Zamanian, Rabinovitch, & Thompson, 2018). As a further point of comparison to the cancer literature, this may be due in part not only to an increase in PMN‐MDSC but a corresponding elevation in PD‐L1 by a neutrophilic subset of immature myeloid cells (Ballbach et al., 2017). Such a cell autonomous effect has been demonstrated already in related disease models, whereby macrophage/monocyte subsets from patients with coronary artery disease express increased PD‐L1, leading to T cell inhibition and a predisposition to reactivation of viral infection (Watanabe et al., 2017). Importantly, however, enthusiasm for novel application of PD‐1/PD‐L1 directed therapy must be approached with caution, as overactivation of T cells, associated especially with dual checkpoint inhibitor blockade, can have deadly consequences (Johnson et al., 2016). Such a concern should not however preclude the study of this drug class further, as current therapies used for treatment of IPF are not without potentially deadly side effects leading to worsened PH and mortality (Shimomura et al., 2019).

As described, myeloid‐derived suppressor cells—particularly the polymorphonuclear subset (PMN‐MDSCs)—act to facilitate immune cell senescence in part through enhanced Arg1 production, primarily described in relation to either cancer (Romano et al., 2018) or autoimmune disease (Wu et al., 2016). The disease context of myeloid‐derived suppressor cells action is thus dependent upon specific T cell subgroup inhibition, illustrated by the changes in phenotype contingent upon Treg or Th17 cell proliferation/activation, that may act in concert with myeloid‐derived suppressor cell signalling (Hoechst, Gamrekelashvili, Manns, Greten, & Korangy, 2011; Ji et al., 2016). For example, in irradiation‐induced pneumonitis/fibrosis, Treg depletion alleviates lung inflammation invoking a see‐saw effect on Th17 population, which is then elevated (Xiong et al., 2015); this has been observed in silica‐induced pulmonary fibrosis, as well (Liu et al., 2010). The effect of the Treg:Th17 ratio is likely more complicated, however, with qualitative as well as quantitative measures contributing to disease progression, leading to seemingly contradictory findings depending on which cell population is targeted (Thakur et al., 2015). Illustrative of this concept is the finding that in patients with IPF, there is an increase in circulating Treg and a decrease in Th17, similar to experimental results from cancer studies, but not those examining autoimmunity (Galati et al., 2014). A compensatory response to overcome an intrinsic defect in Treg function may be responsible, however, as another study demonstrated impaired suppressive capability by Treg from patients with IPF (Kotsianidis et al., 2009).

Similar findings have also been described in PH. For example, in rats exposed to hypoxia—inducing durable PH—those with an increase in Th17 compared to Treg cells developed more severe PH (Li et al., 2018). Conversely, in a clinical study of PAH patients, there was an increase in circulating Treg (an increase in the Treg:Th17 ratio) associated with worsened pulmonary vascular disease (Jasiewicz et al., 2016). Finally, in a patient population with PH related to connective tissue disease, a decrease in Treg population was associated with a worse prognosis and more severe PH (Gaowa et al., 2014). Moreover, the finding was worse in those with a documented depression in FEV1, potentially indicating patients with co‐existing fibrotic lung disease. It is intriguing to speculate, based upon the results of our own study, that this delicate balance between T lymphocyte subpopulations is variably dependent upon PMN‐MDSC associated PD‐1/PD‐L1 activity (Limagne et al., 2016).

To our knowledge, this is the first study of its kind to examine the LysM.Cre‐DTR transgenic mouse construct in the setting of chronic diphtheria toxin exposure (greater than 2–4 weeks), although it is not the first to demonstrate a robust bone marrow cellular response to repetitive doses of diphtheria toxin (Hua, Shi, Shultz, & Ren, 2018; Lee, Qiao, Kinney, Feng, & Shao, 2014), after a period of days to a full week. While diphtheria toxin will affect myeloid‐derived suppressor cells, potentially to an even greater degree than mature circulating myeloid cells, our data are consistent with the previously reported robust response of bone marrow progenitor cell proliferation, and release into circulation, of myeloid‐derived suppressor cells in the setting of rapid turnover due to chronic inflammation (PMID 24789911). Thus, the novelty of our findings is that likely the increase in myeloid cells and therefore myeloid‐derived suppressor cells, was due predominantly to the myelopoietic response of these mice to chronic diphtheria toxin administration.

Conclusions drawn from our study are tempered by several limitations. First, we provide mechanistic insight into prevention of disease only—we make no inferences on treatment of existing Group 3 PH, as the goal of the study was to establish the relevance of PD‐L1 therapy in a robust model of PH secondary to ILD. Future studies will need to be performed to this end. Additionally, interpretation of our model must be informed by the lack of cell specificity in depletion strategy. Namely, we cannot rule out effects on disease progression due to the loss of organ‐specific cellular regulatory elements such a lung interstitial or alveolar macrophages. Also, given that the LysM Cre‐recombinase is expressed by nearly a quarter of pulmonary epithelial cells (McCubbrey et al., 2017), we cannot definitively rule out either an earlier contribution of epithelial cell death to our phenotype, nor can we state that there is not a cell‐autonomous effect of loss of the epithelial lining on development of PH secondary to bleomycin‐induced pulmonary fibrosis. However, given our prior report on abrogation of the PH response in a similar model of disease, by merely blocking accumulation of myeloid‐derived suppressor cells, we do not feel that such a loss of native cell populations is playing a large part in the observed maladaptive pulmonary vascular changes (Bryant et al., 2018). Finally, though less of a weakness of this study than a strong future direction for research, the downstream mechanism of either myeloid‐derived suppressor cells or T lymphocyte action in either fibrotic or vascular lung changes is unknown. Likewise, an upstream cause for elevated PD‐L1 expression by PMN‐MDSC is still a mystery. Such caveats, in our opinion, are not necessary to understand the importance of PD‐L1 targeting though, with safe and readily available existing therapies on the market, in treatment of a disease that currently has none.

## CONFLICT OF INTEREST

The authors have declared no conflicts of interest, except C.E.V. who reports personal fees from Acceleron Pharma, personal fees from Bayer, grants to her institution from Eiger and United Therapeutics, and personal fees (travel, editorial work) from American Thoracic Society, outside the submitted work; Spouse is an employee of CVS Health.

## AUTHOR CONTRIBUTIONS

C.F. designed and performed experiments, analysed data, and wrote the manuscript; Y.L. and M.A.W. performed experiments; M.L.B. and C.E.V. designed experiments and edited the manuscript; L.M.M., B.M., and E.W.S. edited the manuscript; A.J.B. designed and performed experiments, analysed data, and wrote and edited the manuscript.

## DECLARATION OF TRANSPARENCY AND SCIENTIFIC RIGOUR

This Declaration acknowledges that this paper adheres to the principles for transparent reporting and scientific rigour of preclinical research as stated in the *BJP* guidelines for Design & Analysis, Immunoblotting and Immunochemistry, and Animal Experimentation, and as recommended by funding agencies, publishers, and other organizations engaged with supporting research.

## Supporting information

Figure S1. (A) Collagen 1A1 (Col1A1) and (B) Fibronectin RT‐PCR analysis of mRNA expression (relative units) in whole lung tissue from vehicle (Veh) or bleomycin (Blm) exposed mDTR mice treated with or without DT, normalized to 18S. (C) Bronchoalveolar lavage fluid protein concentration (micrograms/milliliter) in treatment groups. (D and E) Quantification per high‐powered field, and images of, Terminal deoxynucleotide transferase‐mediated dUTP nick‐end labelling (TUNEL) staining of murine lung tissue; experimental groups as indicated. n = 5 mice per vehicle group, and 9 mice per bleomycin group. All data are presented as mean ± SEM. P < 0.05 was considered significant

Figure S2. Soluble growth factors and cytokines, analyzed by luminex assay, resulted from whole lungs of LysM.Cre‐DTR (“mDTR”) mice treated either with or without diphtheria toxin (DT) and vehicle (Veh) or bleomycin (Blm) including: (A) G‐CSF, (B) Eotaxin, (C) IL‐1 β, (D) IL‐2, (E) MIP‐2, and (F) RANTES. n = 5 mice per vehicle group and 7 mice per bleomycin group. All data are presented as mean ± SEM. P < 0.05 was considered significant

Figure S3. (A) Western blot for collagen and fibronectin expression in lung homogenates obtained 33 days after vehicle (Veh) or bleomycin (Blm) injection protocol in mice treated with anti‐PD‐L1 antibody (αPD‐L1) or IgG control. Beta‐actin (β‐actin) served as a loading control. Shown are representative samples per condition. (B and C) Quantification per high‐powered field, and images of, Terminal deoxynucleotide transferase‐mediated dUTP nick‐end labelling (TUNEL) staining of murine lung tissue; experimental groups as indicated. n = 6 mice/group, except for immunoblot. All data are presented as mean ± SEM. P < 0.05 was considered significant

Table S1. Complete list of antibodies

Table S2. Patient Characteristics for Myeloid‐Derived Suppressor Cell (MDSC) Analysis*
