# Supplementary figures and images for: Emergency myelopoiesis contributes to immune cell exhaustion and pulmonary vascular remodelling
Source: Br J Pharmacol. 2020 Feb 4;178(1):187–202. doi: 10.1111/bph.14945 (PMC8240454; doi:10.1111/bph.14945)

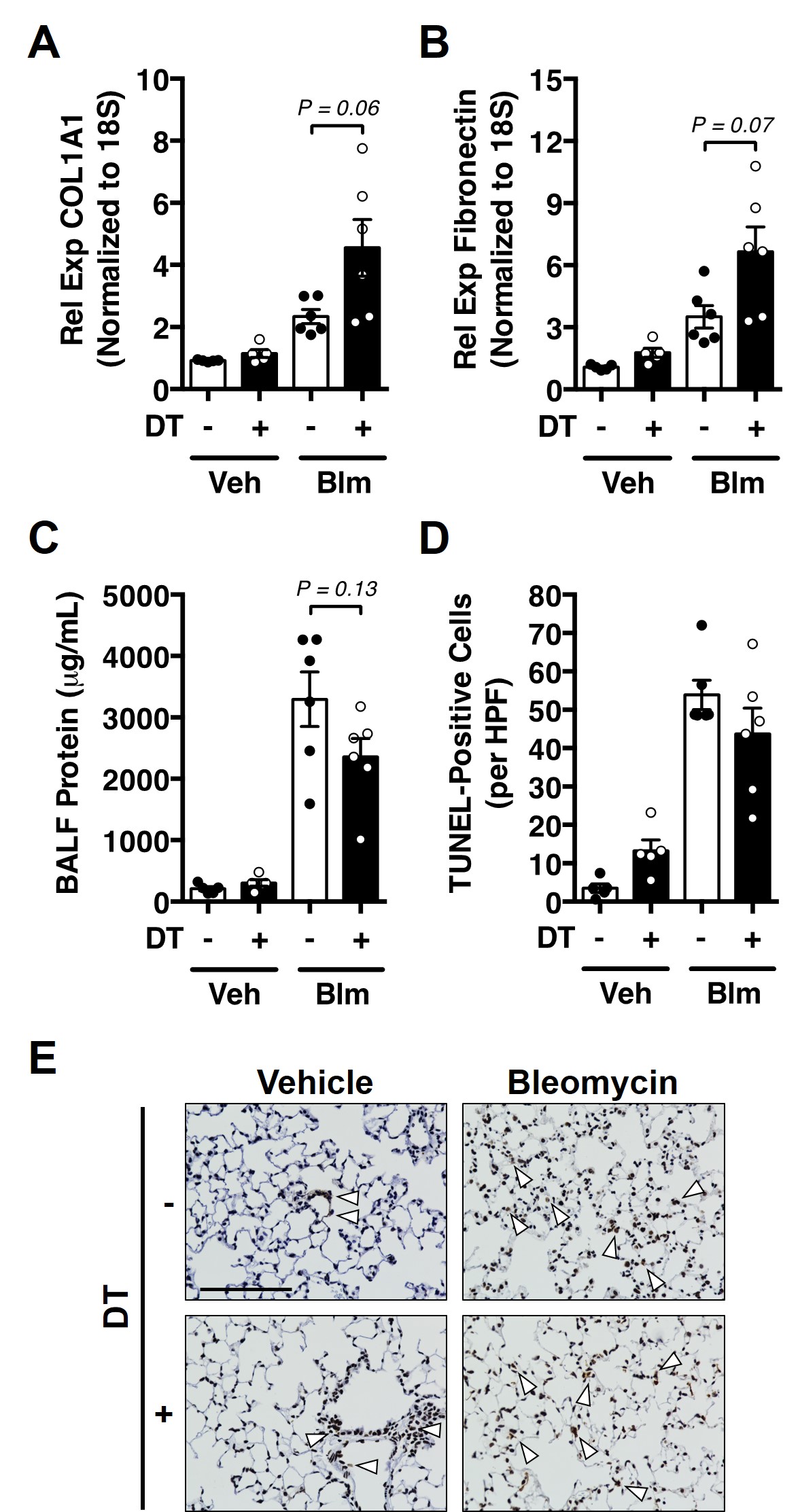

Supplement: Supplementary file 1 — Figure S1. (A) Collagen 1A1 (Col1A1) and (B) Fibronectin RT‐PCR analysis of mRNA expression (relative units) in whole lung tissue from vehicle (Veh) or bleomycin (Blm) exposed mDTR mice treated with or without DT, normalized to 18S. (C) Bronchoalveolar lavage fluid protein concentration (micrograms/milliliter) in treatment groups. (D and E) Quantification per high‐powered field, and images of, Terminal deoxynucleotide transferase‐mediated dUTP nick‐end labelling (TUNEL) staining of murine lung tissue; experimental groups as indicated. n = 5 mice per vehicle group, and 9 mice per bleomycin group. All data are presented as mean ± SEM. P < 0.05 was considered significant [file BPH-178-187-s005.jpg]

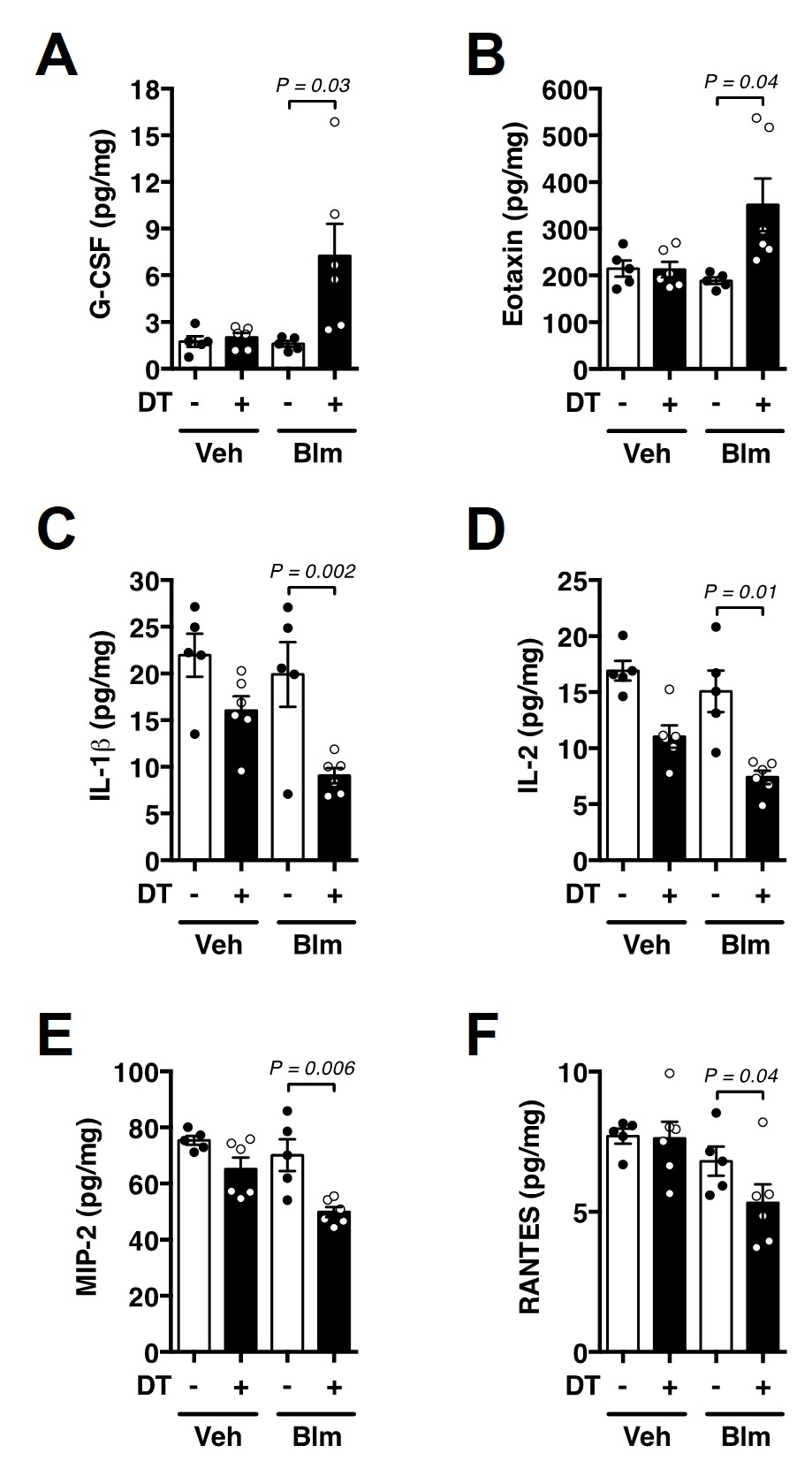

Supplement: Supplementary file 2 — Figure S2. Soluble growth factors and cytokines, analyzed by luminex assay, resulted from whole lungs of LysM.Cre‐DTR (“mDTR”) mice treated either with or without diphtheria toxin (DT) and vehicle (Veh) or bleomycin (Blm) including: (A) G‐CSF, (B) Eotaxin, (C) IL‐1 β, (D) IL‐2, (E) MIP‐2, and (F) RANTES. n = 5 mice per vehicle group and 7 mice per bleomycin group. All data are presented as mean ± SEM. P < 0.05 was considered significant [file BPH-178-187-s004.jpg]

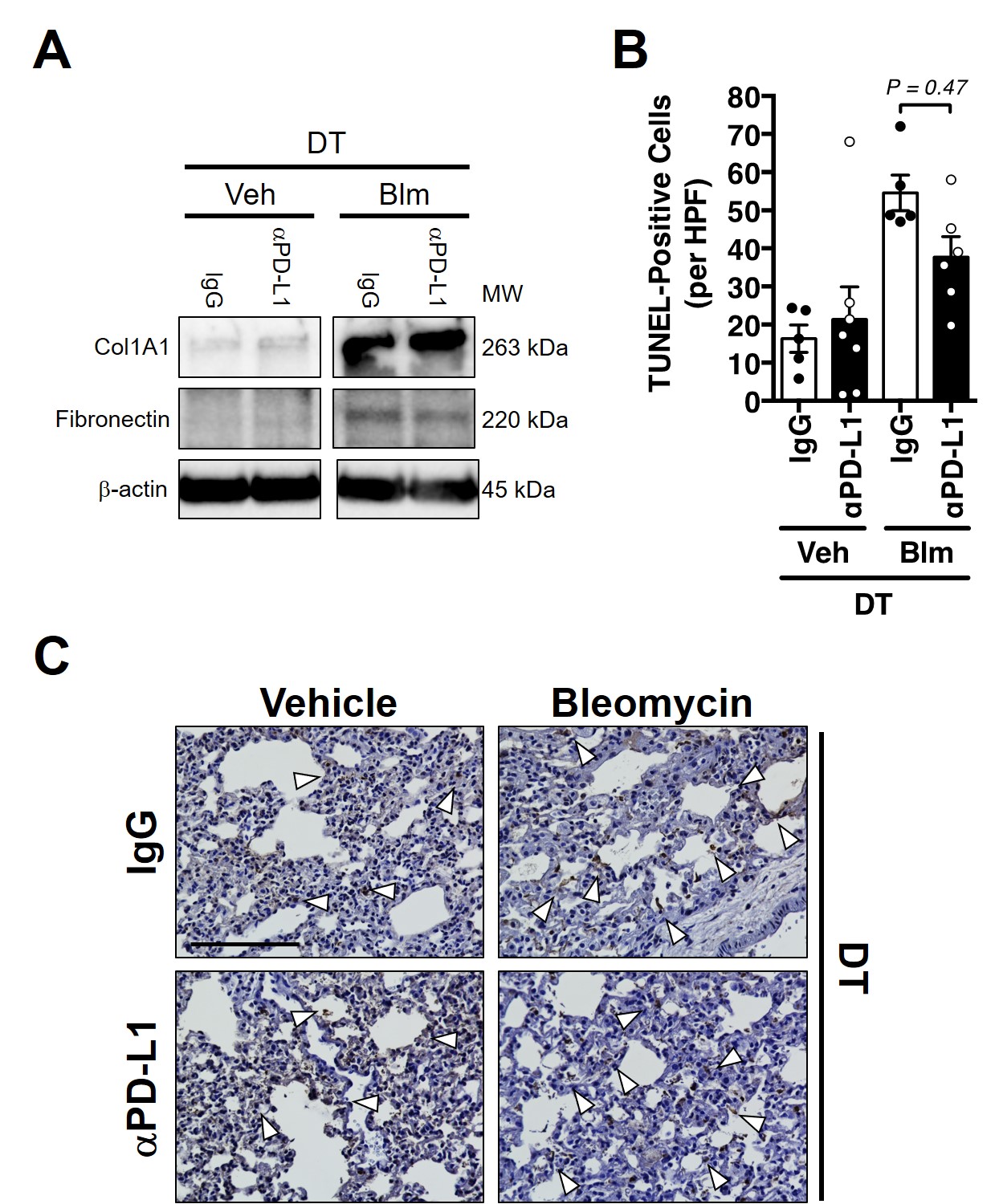

Supplement: Supplementary file 3 — Figure S3. (A) Western blot for collagen and fibronectin expression in lung homogenates obtained 33 days after vehicle (Veh) or bleomycin (Blm) injection protocol in mice treated with anti‐PD‐L1 antibody (αPD‐L1) or IgG control. Beta‐actin (β‐actin) served as a loading control. Shown are representative samples per condition. (B and C) Quantification per high‐powered field, and images of, Terminal deoxynucleotide transferase‐mediated dUTP nick‐end labelling (TUNEL) staining of murine lung tissue; experimental groups as indicated. n = 6 mice/group, except for immunoblot. All data are presented as mean ± SEM. P < 0.05 was considered significant [file BPH-178-187-s003.jpg]
